# Prognostic Association of YB-1 Expression in Breast Cancers: A Matter of Antibody

**DOI:** 10.1371/journal.pone.0020603

**Published:** 2011-06-10

**Authors:** Adele G. Woolley, Michael Algie, Weini Samuel, Rhodri Harfoot, Anna Wiles, Noelyn A. Hung, Puay-Hoon Tan, Peter Hains, Valentina A. Valova, Lily Huschtscha, Janice A. Royds, David Perez, Han-Seung Yoon, Scott B. Cohen, Phillip J. Robinson, Boon-Huat Bay, Annette Lasham, Antony W. Braithwaite

**Affiliations:** 1 Department of Pathology, University of Otago, Dunedin, New Zealand; 2 Children's Medical Research Institute and Faculty of Medicine, University of Sydney, Westmead, Australia; 3 Department of Molecular Medicine and Pathology, University of Auckland, Auckland, New Zealand; 4 Department of Anatomy, Yong Loo Lin School of Medicine, National University of Singapore, Singapore, Singapore; 5 Department of Pathology, Singapore General Hospital, Singapore, Singapore; 6 Department of Oncology, Dunedin Public Hospital, Dunedin, New Zealand; University of Pennsylvania, United States of America

## Abstract

The literature concerning the subcellular location of Y-box binding protein 1 (YB-1), its abundance in normal and cancer tissues, and its prognostic significance is replete with inconsistencies. An explanation for this could be due in part to the use of different antibodies in immunohistochemical and immunofluorescent labeling of cells and tissues. The inconsistencies could also be due to poor resolution of immunohistochemical data. We analyzed two cohorts of breast tumours for both abundance and subcellular location of YB-1 using three different antibodies; two targeting N-terminal epitopes (*AB-* ***a*** and *AB-* ***b***) and another (*AB-* ***c***) targeting a C-terminal epitope. We also investigated stress-induced nuclear translocation of YB-1 in cell culture. We report that both *AB-* ***a*** and *AB-* ***c*** detected increased YB-1 in the cytoplasm of high-grade breast cancers, and in those lacking estrogen and progesterone receptors; however the amount of YB-1 detected by *AB-* ***a*** in these cancers is significantly greater than that detected by *AB-* ***c***. We confirm our previously published findings that *AB-* ***b*** is also detecting hnRNP A1, and cannot therefore be used to reliably detect YB-1 by immunohistochemistry. We also report that *AB-* ***a*** detected nuclear YB-1 in some tumour tissues and stress treated cells, whereas *AB-* ***c*** did not. To understand this, cancer cell lines were analyzed using native gel electrophoresis, which revealed that the antibodies detect different complexes in which YB-1 is a component. Our data suggest that different YB-1 antibodies show different staining patterns that are determined by the accessibility of epitopes, and this depends on the nature of the YB-1 complexes. It is important therefore to standardize the protocols if YB-1 is to be used reproducibly as a prognostic guide for different cancers.

## Introduction

Y-box binding protein-1 (YB-1, P67809) is a member of the cold-shock superfamily and plays a role in multiple biological processes including cell proliferation, DNA repair, translation and transcription (reviewed in [1], [2], [3]). Despite being able to function as a transcription factor, >90% of YB-1 is located in the cytoplasm [1] where it binds RNA and regulates translation [4], [5]. Nuclear translocation of YB-1 has been reported to occur during the G_1_ to S phase transition of the cell cycle [6] and in response to a range of stressors including ultraviolet (UV) radiation [7], [8] and DNA damaging agents, such as cisplatin [8], [9] and mitomycin C [10]. As tumour cells are thought to be under constant stress due to sequential mutations, the significance of nuclear YB-1 in cancer has been the focus of ongoing investigation.

Early immunohistochemical observations showed that YB-1 protein is elevated in ∼75% of breast cancers [11]. This was subsequently extended to a wide range of common human cancers, including cancers of the prostate [12], lung [13], skin [14], bone [15], and others [16], [17], [18]. However, there is disagreement as to whether nuclear YB-1 is a significant prognostic factor and there are discrepancies in the literature as to whether YB-1 is present in normal tissues. For example, immunohistochemical studies report an absence of YB-1 staining in normal breast tissue [19] and melanocytes [14] but clear evidence of both nuclear and cytoplasmic staining in tumour tissues with elevated levels of both being associated with tumour progression. Increased nuclear YB-1 has also been reported to correlate with lymph node metastasis in patients with non-small cell carcinoma [20], but this correlation was not reported by others [13]. Nuclear YB-1 staining has also been associated with increased expression of multidrug resistance 1 (MDR1) in patients with poor prognosis [11], [21]. In other reports, increased cytoplasmic YB-1 was associated with poor patient prognosis where nuclear YB-1 was rarely detected (in <2% of tumours) [22].

**Figure 1:**
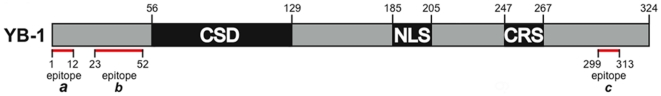
Linear representation of YB-1. YB-1 contains a highly conserved cold shock domain (CSD); a nuclear localization signal (NLS); a cytoplasmic retention signal (CRS); red bars indicate the epitopes (***a***–***c***) for YB-1 antibodies.

One possible explanation for these differential immunostaining patterns is that the antibodies used in the above studies have different immunoreactive properties. The majority of antibodies used in these studies are generated to either residues within epitope ***a*** (Figure 1) [11], [19], [21], or to residues 299–313 within epitope ***c*** [12], [13], [18], [22], [23], [24] and are polyclonal antibodies raised in rabbit resulting in an inherent variability in immunoreactivity. If true, the prognostic significance of YB-1 immunostaining would therefore be highly antibody dependent and such variations would make the development of an YB-1 based prognostic marker difficult.

To test this hypothesis, we examined two breast cancer cohorts with 3 antibodies whose epitopes are identified in Figure 1. Our studies show that *AB* ***-b*** is of little prognostic value overall, due to cross-reactivity with hnRNP A1 [25]. On the other hand *AB* ***-a*** and *AB* ***-c*** both have significant prognostic value, as their immunoreactivities correlated with both increasing grade and the absence of estrogen and progesterone receptors (ER/PR negative). However *AB* ***-a*** appeared to be more sensitive at detecting a prognostic association. We also found that *AB* ***-a*** detected nuclear YB-1, while *AB* ***-c*** did not, both in tumours and in cells treated with UV and cisplatin. We propose that this differential immunoreactivity is due to protein-protein interactions rendering the epitope required for *AB-* ***c*** binding unavailable. Our findings bear relevance to the numerous studies that aim to establish YB-1 as a prognostic indicator and may impact on the development of a YB-1 based prognostic screen**.**

## Materials and Methods

### Clinical samples

Breast cancer biopsies from Dunedin Public Hospital, New Zealand, obtained prior to treatment, (n = 90; Table 1) were examined. Normal breast tissue was obtained from 10 reduction mammoplasties, together with normal adjacent tissue (≥10 mm from the malignant tissue). Additionally, two separate tissue microarrays (TMAs) were obtained from the Singapore General Hospital (n = 206, Table 2).

**Table 1:** Clinical and Pathological Characteristics of the NZ Cohort.

Number of patients		90
Median age (range) (years)		58 (28–86)
Pathological stage at diagnosis		
	Stage 1	13
	Stage 2	67
	Stage 3	10
24 month survival (%)		
	Stage 1	100
	Stage 2	91
	Stage 3	61
Histological type [n (%)]		
	Ductal	83 (82)
	Lobular	6 (7)
	Tubular	1 (1)
Histological grade [n (%)]		
	Grade 1	32 (35)
	Grade 2	32 (35)
	Grade 3	28 (30)
Lymph node status [n (%)]		
	Negative	40 (44)
	Positive	50 (56)
Estrogen Receptor status [n (%)]		
	Negative	22 (24)
	Positive	68 (76)
Progesterone Receptor status [n (%)]		
	Negative	39 (43)
	Positive	51 (57)
Subtype [n (%)]		
	Luminal	72 (80)
	Luminal A	49 (54)
	Basal-like	8 (9)
	Her2+	10 (11)

**Table 2:** Clinical and Pathological Characteristics of the Singapore Cohort.

Number of patients		206
Median age (range) (years)		50 (24–85)
Ethnicity		
Chinese [n (%)]		176 (85)
Malay		16 (8)
Indian		8 (4)
Other		6 (3)
Pathological stage at diagnosis [n (%)]		
	Stage 1	42 (20)
	Stage 2	119 (58)
	Stage 3	35 (17)
24 month survival (%)		
	Stage 1	100
	Stage 2	96
	Stage 3	93
Histologic type [n (%)]		
	Ductal	185 (89)
	Lobular	14 (5)
	Tubular	7 (3)
Histologic grade [n (%)]		
	Grade 1	42 (20)
	Grade 2	82 (40)
	Grade 3	82 (40)
Lymph node status [n (%)]		
	Negative	108 (52)
	Positive	98 (48)
Estrogen Receptor status [n (%)]		
	Negative	24 (12)
	Positive	182 (88)
Progesterone Receptor status [n (%)]		
	Negative	20 (10)
	Positive	186 (90)
Subtype		no data

### Ethics statement

Written consent for samples used in the New Zealand cohort was obtained from patients and the use of these samples was approved by the Otago Ethics Committee, Ministry of Health, New Zealand Government, CPD 02/01 and also by the Multi-region Ethics Committee, Ministry of Health, New Zealand Government, MEC/07/05/065. Written consent for the Singapore TMAs was obtained from patients and use of the material was approved by the Institutional Review Board of the Singapore General Hospital.

### Antibodies

Three rabbit polyclonal antibodies were used in this study. *AB-* ***a*** was affinity purified using the immunizing peptide MSSEAETQQPPA, as previously described [16], [25]. *AB-* ***c*** was affinity purified using the immunizing peptide CDGKETKAADPPAENS (residues 299–313, epitope ***c***, Figure 1, as previously described [25]). Both of these antibodies were affinity purified with a column containing the YB-1 peptide conjugated to Thiopropyl-Sepharose 6B gel (column prepared by Mimotopes). *AB-* ***b*** is a commercially available antibody (Abcam ab12148) targeting residues 23–52 (Figure 1, epitope ***b***). A mouse monoclonal to hnRNP A1 (Abcam, ab5832, clone 9H10,) antibody was used in the immunofluorescence experiments. The β-tubulin mouse monoclonal antibody (E7) used as a loading control in Western blotting was obtained from the Developmental Studies Hybridoma Bank (DSHB), Iowa.

### Immunohistochemistry (IHC)

Sections fixed in neutral-buffered-formalin and embedded in paraffin wax were processed using a standard citrate buffer antigen-retrieval protocol. Three rabbit polyclonal primary antibodies were used to detect YB-1, (see above). These were diluted in 1%BSA in PBS as follows: *AB-* ***a***; 1∶1200, *AB-* ***b***; 1∶4000, *AB-* ***c***; 1∶1000 and incubated overnight at 4°C. Detection of the primary antibody carried out using the EnVision™+ Dual Link system (Dako) according to the manufacturer's protocol. Specimens were counterstained in Gills haematoxylin and mounted in Entellan (ProSciTech). Staining pattern and intensity was visualized using a Zeiss Axioplan compound microscope, and photographed with a SPOT-RT CCD camera (Diagnostic Instruments).

### Immunohistochemistry Assessment

**Figure 2:**
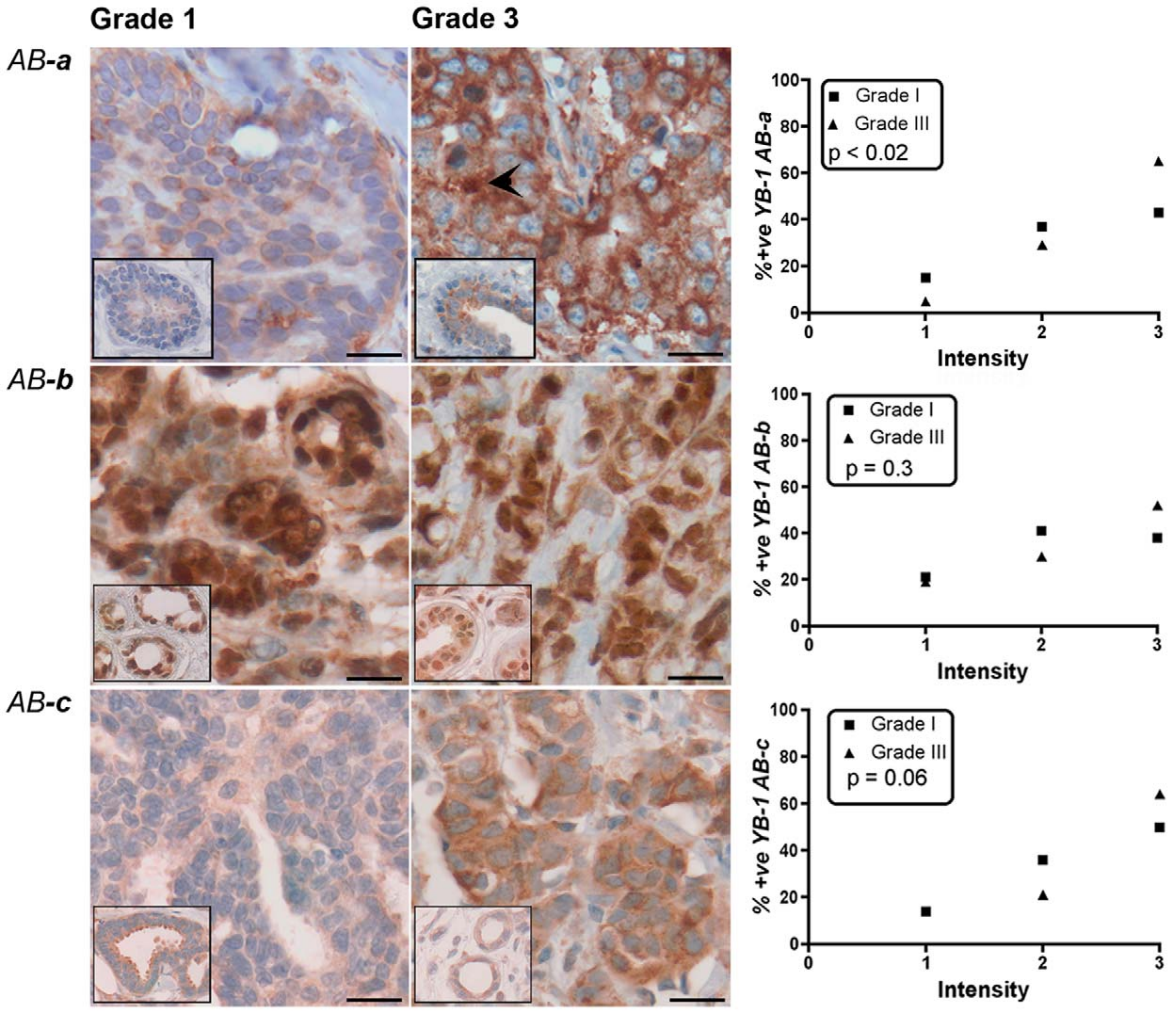
Levels of YB-1 are increased in grade 3 breast tumours as detected by *AB-a*, but not other antibodies. IHC was carried out on formalin-fixed paraffin-embedded breast tumours (n = 90) from the New Zealand cohort using three antibodies targeting different epitopes (***a–c***) and compared to adjacent normal tissue (insets). Staining patterns and levels of expression are shown for each antibody. Differences in staining intensity as detected by *AB-* ***a*** were significant (p<0.02) whereas those detected by *AB-* ***c*** were not (p = 0.06). Nuclear YB-1, as detected by *AB-* ***a*** was evident in <3% of cases (arrowhead, upper middle panel). *Scale bars 10* *µm.*

100 cells in each specimen were scored according to the presence and intensity of staining. Negative staining was scored as zero, weak as one, moderate as two and strong as three. The intensity of staining within the tumour was compared to at least three regions of adjacent normal tissue, as shown in the insets of Figure 2. Identification and assessment of immunohistochemical staining of diagnostic breast tissue was carried out in consultation with registered pathologists.

### Cells and cell culture

Established cell lines A549, MCF-7, and T47D were obtained from the CMRI cell line repository and were validated for authenticity by CellBank Australia using short-tandem-repeat profiling. A549 cells were cultured at 37°C, 5% CO_2_ in Dulbecco's Modified Eagle's Medium (DMEM) (Invitrogen) supplemented with 10% v/v fetal bovine serum (FBS). The breast cancer cell lines MCF-7 and T47D were cultured at 37°C, 10% CO_2_ in DMEM without phenol red (Invitrogen) supplemented with 10% v/v FBS and insulin (Sigma, 10 µg/ml). Primary cell lines were related back to original donor tissues. IIICF/c, Fre16 and melanocytes were cultured at 37°C, 5% CO_2_ in Dulbecco's Modified Eagle's Medium (DMEM) (Invitrogen) supplemented with 10% v/v fetal bovine serum (FBS, JRH). Bre56 and Kre36 cells were cultured in MCDB 170 medium (Invitrogen) and defined keratinocyte-serum free medium (Invitrogen) respectively. Both were grown at 37°C, 5% CO_2_.

### Cell stress treatment

Cells (2×10^4^ cells per well) were seeded into chamber slides, treated as indicated, and incubated at 37°C for 24 h. Cisplatin (Sigma P4394) was prepared just before use as a stock solution in DMSO at a concentration of 10 mM (1000× working concentration). Cells were treated with 10 µM cisplatin for 24 h. For UV irradiation, the culture medium was removed and the cells were covered with PBS. Cells were irradiated at 10 mJ/cm2 using a Bio-link Crosslinker (Vilber Lourmat, France) then further incubated at 37°C.

### Immunoprecipitation

Cell lysates were immunoprecipated with both antibodies (*AB-* ***a*** and *AB-* ***b***) as previously described [25]; however DNA-based purification was omitted.

### Mass Spectrometry and phosphopeptide enrichment

Protein bands were excised from a Coomassie-stained gel, destained and subjected to trypsin digest as previously described [25]. Following digestion, phosphopeptides were purified as described [26] and then analysed on a Thermo Velos Orbitrap mass spectrometer coupled to a Dionex Ulitmate 3000 HPLC system. Peptides were loaded onto a pre-column (Dionex 300 µm×5 mm, C18, 5 µm) and separated on a 12 cm×100 µm column packed with ReproSil-Pur 120 C18-AQ 3 µm resin (Dr. Maisch GmbH) running at 250 nl/min. Samples were loaded in 0.5% (v/v) formic acid in water and separated over a 35 min linear gradient of 0% (v/v) acetonitrile to 32% (v/v) acetonitrile. The percentage acetonitrile was linearly increased to 55% (v/v) over the next 5 min and then to 90% (v/v) over a one min period. The column was re-equilibrated prior to each sample. The initial MS scan (350–2000 m/z) was performed in the Orbitrap with the resolution set to 60,000. Following this scan, MS/MS of up to the top 20 peptides was performed in the linear ion trap with the normalised collision energy set to 35. Data were searched using Mascot 2.3.0 and the SWISS-PROT database (56.6) limited to human (20413 sequences) with the precursor mass set to 10 ppm and MS/MS tolerance at 0.6 Da. Modifications included deamidation (N,Q), methionine sulphoxide and phosphorylation (S,T,Y). All phosphopeptides were manually analysed for correct site assignment.

### Immunofluorescent cytochemistry

For slide preparation, medium was removed from wells, washed with PBS, and fixed with paraformaldehyde (2% v/v). They were then permeabilized with PBS/0.5% v/v Triton X-100 and after further washing, incubated with primary antibodies (YB-1, 1∶1000; hnRNP A1, 1∶1000) in antibody solution (2% w/v BSA, 0.1% w/v NaN3, 0.2% w/v cold fish gelatin, in TBS/0.1% v/v Triton X-100) and incubated for 1 h. Slides were then stained with Alexa-Fluor 488 goat anti-rabbit secondary antibody (Invitrogen 1∶2000) in the presence or absence of the nuclear marker 4′-6-Diamidino-2-phenylindole (DAPI; Invitrogen). Cells were visualized with a Leica Axioplan upright microscope equipped for epifluorescence and photographed with a SPOT-RT Slider cooled CCD camera (Diagnostic Instruments).

### Confocal immunofluorescence microscopy

Formalin-fixed paraffin-embedded tumour sections were processed as above and residual aldehydes quenched with 0.1 M glycine in PBS. Following antigen retrieval, rabbit polyclonal primary antibodies to epitopes ***a*** and ***c*** were used to label YB-1 and detected with an anti-rabbit IgG Alexa Fluor 488 secondary antibody (Invitrogen, 1∶2000). Labelling was visualised by confocal analysis using a Zeiss LSM 510 confocal laser scanning microscope. Multiple z-stacks were collected for each sample (0.47 µm optical dissection).

### SDS-PAGE and Immunoblotting

Cells were harvested as previously described [25] and the protein content of each sample was determined by BCA protein assay and standardized accordingly. Equal volumes of samples and DSL loading buffer were denatured and loaded on a 10–12% SDS–PAGE. Following electrophoresis, protein extracts were transferred to PVDF membrane (Amersham). Immunoblotting experiments were carried out with YB-1 antibodies as described previously at a dilution of 1∶1500 and incubated overnight at 4°C. β-tubulin (E7, DSHB) was used as a loading control at a dilution of 1∶20,000. Detection was carried out according to standard procedures and bands visualized using the WesternBreeze™ chemiluminescent system (Invitrogen).

### Nuclear Fractionation and Native Gel Electrophoresis

A549 cells were cultured as above and after harvesting were fractionated into cytoplasmic and nuclear compartments as previously described [25]. Blue native polyacrylamide gel electrophoresis (BN-PAGE) was performed using the NativePAGE™ Novex Bis-Tris system as outlined in the user manual (Invitrogen). Immunoblotting was done as above. β-Tubulin antibody (clone E7, Developmental Studies Hybridoma bank, University of Iowa, USA, 1∶2000) was used as a nuclear fractionation marker.

### Statistical Analysis

χ^2^-Test or Fisher′s Exact tests were used as appropriate. p values ≤0.05 were considered statistically significant. Calculations were performed using STATA Version 9.1 (StataCorp) or GraphPad Prism Version 4.0 b software.

## Results

### Characterisation of antibodies

Antibodies to epitopes ***a*** and ***c*** were generated based on published sequences [16], [27], (also see methods), and the specificity of both antibodies has been confirmed by mass spectrometry sequencing [25]. Consistent with this, both antibodies detect a single protein species of ∼49 kDa on SDS-PAGE which is markedly diminished by treatment with two distinct YB-1 specific siRNAs in four different cell lines ([25], A549 and MCF-7; data for other cell lines not shown). The third antibody (epitope ***b***, Abcam ab12148) is used in a public tumour database (http://www.proteinatlas.org). This antibody detects two protein species that migrate at ∼49 kDa and ∼37 kDa, the latter of which was sequenced and identified as hnRNP A1 [25]. Following knockdown of YB-1 (Figure S1) we observed that hnRNP A1 is located in the nucleus whereas YB-1 is abundant in the cytoplasm. However, hnRNP A1 is known to be present in both compartments [28], making the immunostaining produced by the antibody difficult to interpret. This cross-reactivity was discovered once the Dunedin cohort had been analysed and our analyses found that *AB-* ***b*** did not correlate with tumour grade or ER/PR status. Representative images from this work on the Dunedin cohort are included alongside those from the other two antibodies to provide contrast. However, they are not discussed further, as it is not possible to reliably distinguish between YB-1 and hnRNP A1.

### YB-1 staining intensity correlates with tumour grade

To determine whether all YB-1 antibodies showed the same staining pattern and were similarly associated with prognosis, we examined two cohorts of breast tumours comprising different grades to evaluate YB-1 abundance in relation to immediately adjacent normal tissue. One cohort (n = 90) is from New Zealand (clinical details in Table 1) and the other, larger cohort (n = 206) involving a different racial mix, is from Singapore (clinical details in Table 2). Grade 2 tumours were excluded from both cohorts as recent gene expression profiling has revealed that these tumours do not have a distinct genetic profile, but have comparable genetic profiles and clinical outcomes to either grade 1 or 3 tumours [29].

Examples of IHC results from the NZ cohort are shown in Figure 2. The intensity of YB-1 as detected with *AB-* ***a*** (top panels) is clearly higher in the grade 3 tumours compared to grade 1 tumours when normalized to the adjacent normal tissue (insets). We did not find any instances of negative (zero) staining. Further examination showed considerable heterogeneity in staining intensity in both grade 1 and grade 3 tumours compared to adjacent normal tissue. However, quantitative analysis of these data revealed that generally a greater proportion of grade 3 tumours had higher levels of YB-1 than grade 1 tumours (Figure 2, p<0.02; Fisher's exact). A similar staining pattern and trend of increased levels of YB-1 in grade 3 tumours was observed with *AB-* ***c*** (Figure 2, bottom panels), but did not reach significance (Figure 2, p = 0.06; Fisher's exact).

**Figure 3:**
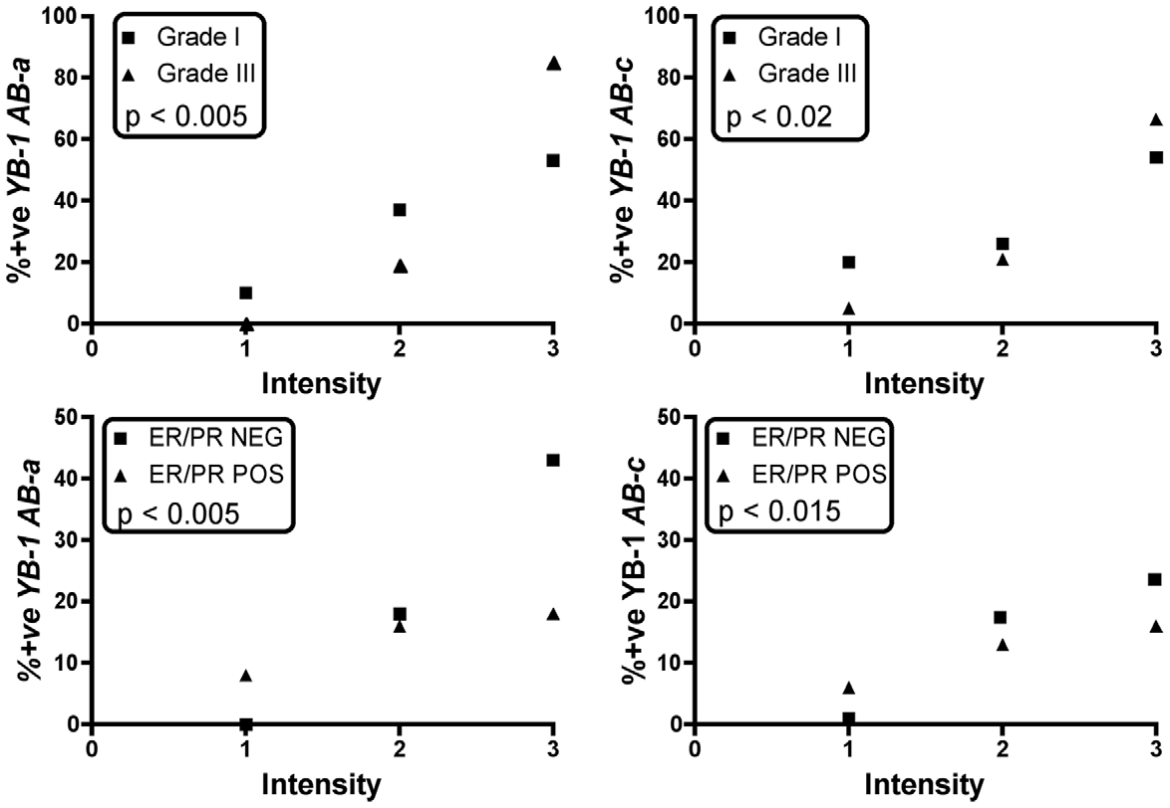
Both *AB-a* and *AB-c* detect increased levels of YB-1 in the larger Singapore cohort. TMAs with samples from 206 breast tumours were stained with *AB-* ***a*** and *AB-* ***c*** and analysed for YB-1 intensity and location. The statistical analysis of this is shown for TMAs on the basis of grade (upper panels) and ER/PR status (lower panels). The correlation of *AB-* ***a*** to tumor grade was highly significant, (χ^2^ = 35.95, p<0.005); that of *AB* ***-c*** was less significant (χ^2^ = 8.623, p<0.02). The correlation of *AB-* ***a*** to ER/PR negativity was highly significant, (χ^2^ = 40.71, p<0.005); that of *AB* ***-c*** was less so (χ^2^ = 28.17, p<0.015).

To determine whether differences in YB-1 antibody staining were also observed in the larger Singapore breast cancer cohort, a similar YB-1 abundance analysis by tumour grade was carried out with *AB-* ***a*** and *AB-* ***c*** (Figure 3, upper panels). In this case YB-1 abundance detectable with *AB-* ***a*** showed a high correlation with tumour grade (χ^2^ = 35.95, p<0.005); similarly, YB-1 detectable with *AB* ***-c*** showed a significant trend with respect to tumour grade, although it was 2 orders of magnitude less significant (χ^2^ = 8.623, p<0.02).

### YB-1 staining intensity is highest in ER/PR negative tumours

**Figure 4:**
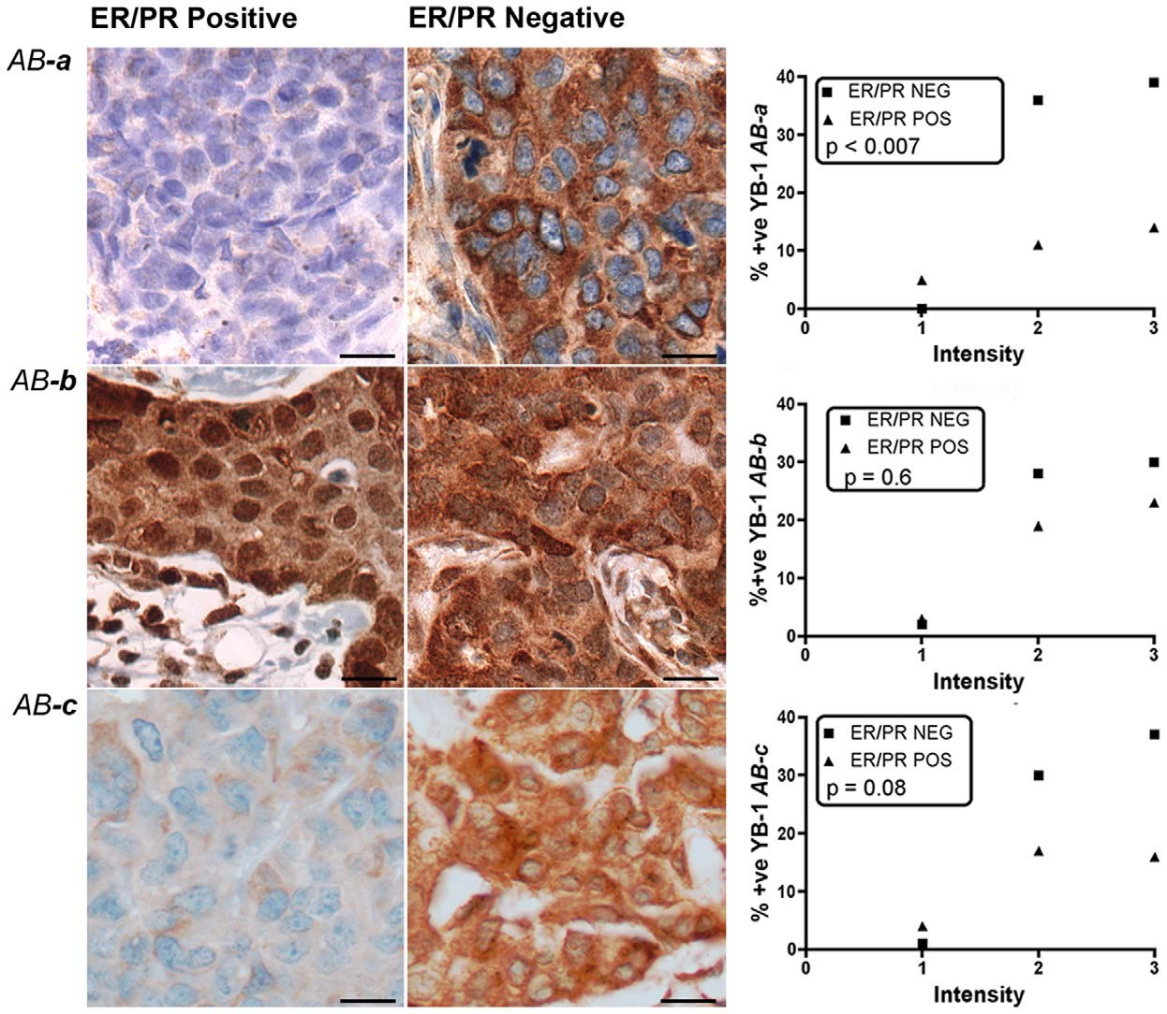
Levels of YB-1 are increased in ER/PR negative breast tumours as detected by *AB-a*, but not other antibodies. IHC was carried out on formalin-fixed paraffin-embedded tumours (n = 90) from the New Zealand cohort using three antibodies targeting different epitopes (***a***–***c***). Staining patterns and levels of expression are shown for representative sections for each antibody: ER/PR positive tumours are represented in the left hand column; ER/PR negative tumours in the central column. Differences in staining intensity as detected by *AB-* ***a*** were significant (p<0.007) whereas those detected by *AB-* ***c*** were not (p = 0.08). *Scale bars 10* *µm.*

Gene expression analysis studies [30], [31] have been used to subtype breast tumours. Of these, tumours that are ER/PR negative include the more aggressive basal-like and ERBB2 (HER2)^+^ tumours. We therefore investigated whether the detectable levels of YB-1 were significantly different in ER/PR negative tumours with each of the YB-1 antibodies. In the NZ cohort, as for tumour grade, we found a greater proportion of ER/PR negative tumours had the strongest YB-1 staining as detected by *AB-* ***a***, which reached a high level of significance over the cohort (Figure 4, p<0.007; Fisher's exact). The staining intensity with *AB-* ***c*** did not reach statistical significance (Figure 4, p = 0.08; Fisher's exact). When the Singapore cohort was examined with *AB-* ***a*** and *AB-* ***c*** (Figure 3, lower panels), both showed more intense staining in the ER/PR negative group, but as was found for tumour grade, the relationship was much stronger with *AB-* ***a*** (χ^2^ = 40.71, p<0.005) than with *AB-* ***c*** (χ^2^ = 28.17, p<0.015).

### Heterogeneous YB-1 expression in normal breast tissue with AB-a and AB-c

**Figure 5:**
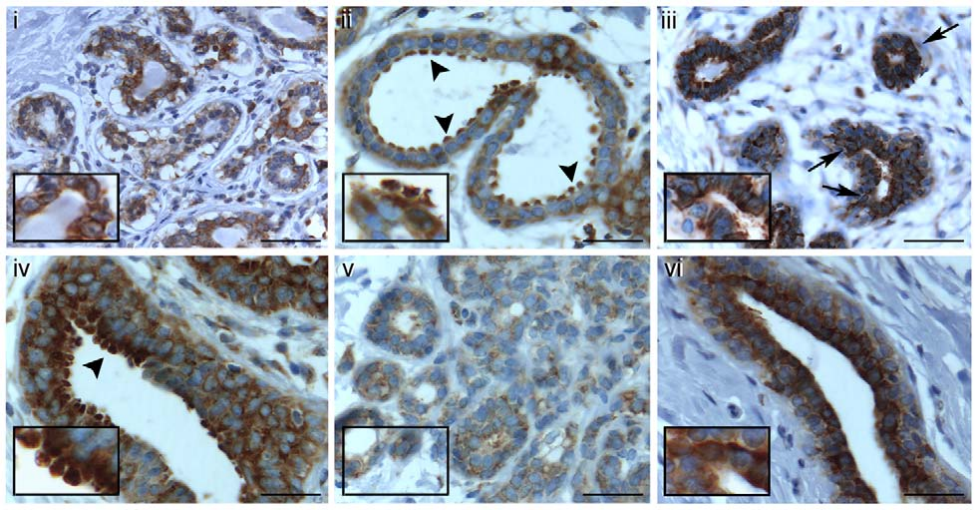
Heterogeneous expression of YB-1 in normal breast tissue. Cytoplasmic and perinuclear YB-1 is detected in both normal lobular (i, iii and v) and ductal (ii, iv and vi) tissue. Insets show staining at a higher magnification. Arrowheads (panels ii and iv) depict high levels of YB-1 in columnar alterations with prominent apical snouts and secretions (CAPSS). *Scale bars 20* *µm*.

Given the heterogeneity of YB-1 staining intensity observed in the tumours and adjacent normal tissue, we investigated non-pathological breast tissue from reduction mammoplasties to see if a similar pattern was evident. Staining with both *AB-* ***a*** (Figure 5) and *AB-* ***c*** (data not shown) showed that YB-1 was present in all of the reduction mammoplasties analysed. This staining was heterogeneous and was particularly intense in cells with columnar alterations of lobules with prominent apical snouts and secretions (CAPSS; arrowheads in Figure 5, ii and iv). Thus the heterogeneity of YB-1 expression is a normal feature of breast tissue. Several normal and cancer cell lines were also examined in which we found YB-1 to be expressed in all cases (Figure S2). Although the levels varied there was no marked difference between normal breast epithelial and breast cancer cell lines.

### Nuclear YB-1 is detected with AB-a in breast cancer tissue but not with AB-c

**Figure 6:**
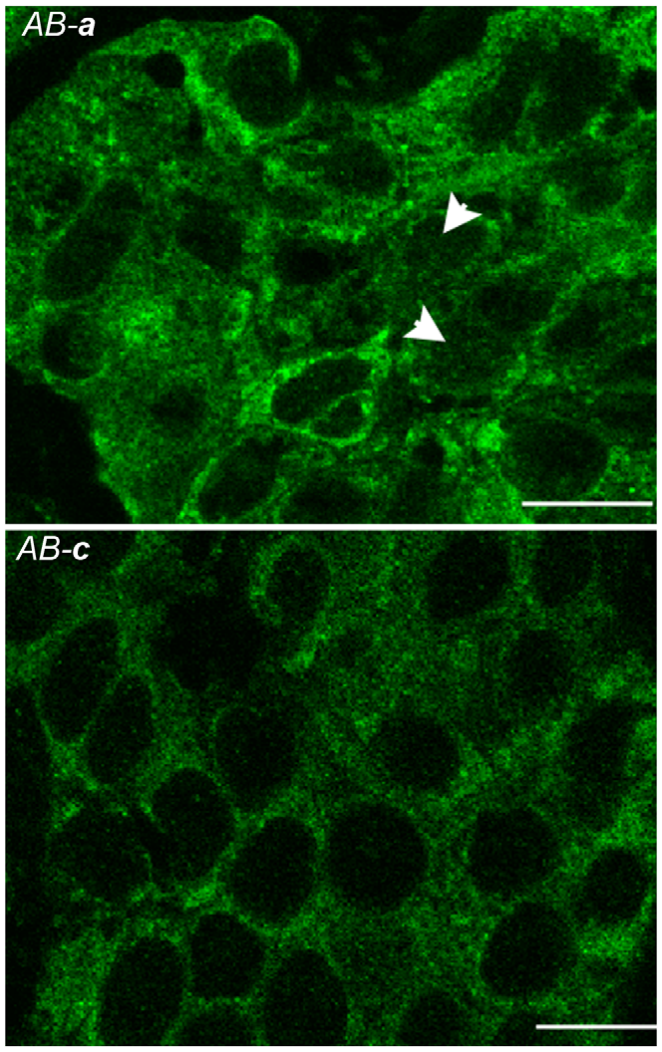
*Ab-a* but not *Ab-c* detects both increased perinuclear cytoplasmic staining and punctate nuclear staining of YB-1 in grade 3 breast tumours. YB-1 in breast tumour sections were labelled with either *Ab-**a*** or *Ab-**c*** and visualized with Alexa Fluor 488 secondary antibody prior to confocal analysis. Representative mid z-stack sections are shown for each of the antibodies. Arrowheads show punctate nuclear staining not detectable by light microscopy. *Scale bars 5.0* *µm.*

During the above experiments subcellular localization of YB-1 was also determined. In almost every case YB-1 was found to be cytoplasmic with antibodies *AB-* ***a*** and *AB-* ***c***, irrespective of which tumour cohort was examined. We found only 3 tumour sections where nuclear YB-1 was apparent with *AB-* ***a*** (eg, see arrowhead Figure 2, grade 3 tumour, *AB-* ***a***). Because conventional IHC is carried out on relatively thick (5 µm) sections we wanted to determine whether this apparent nuclear staining detected with *AB-* ***a*** was real, or due to the plane of section. We therefore imaged the tumour samples with confocal immunofluorescence microscopy using a 0.47 µm optical slice. Results show limited punctate staining in the nucleus with *AB-* ***a*** (Figure 6, upper panel) that is not discernible using conventional microscopy. However, increased perinuclear staining is evident with this antibody (Figure 6, upper panel) so it is possible that such staining could be misinterpreted as nuclear staining at the light microscopy level. Cytoplasmic YB-1 as detected with *AB-* ***c*** was less intense, and there was no evidence of nuclear staining (Figure 6, lower panel). Given the demonstrated specificity of the antibodies, these results suggest that *AB-* ***a*** is detecting a subset of YB-1 that is present, albeit at relatively low levels, within the nucleus that is not detected by *AB-* ***c***.

### Stress-induced nuclear translocation of YB-1 is detected only with antibody AB-a

**Figure 7:**
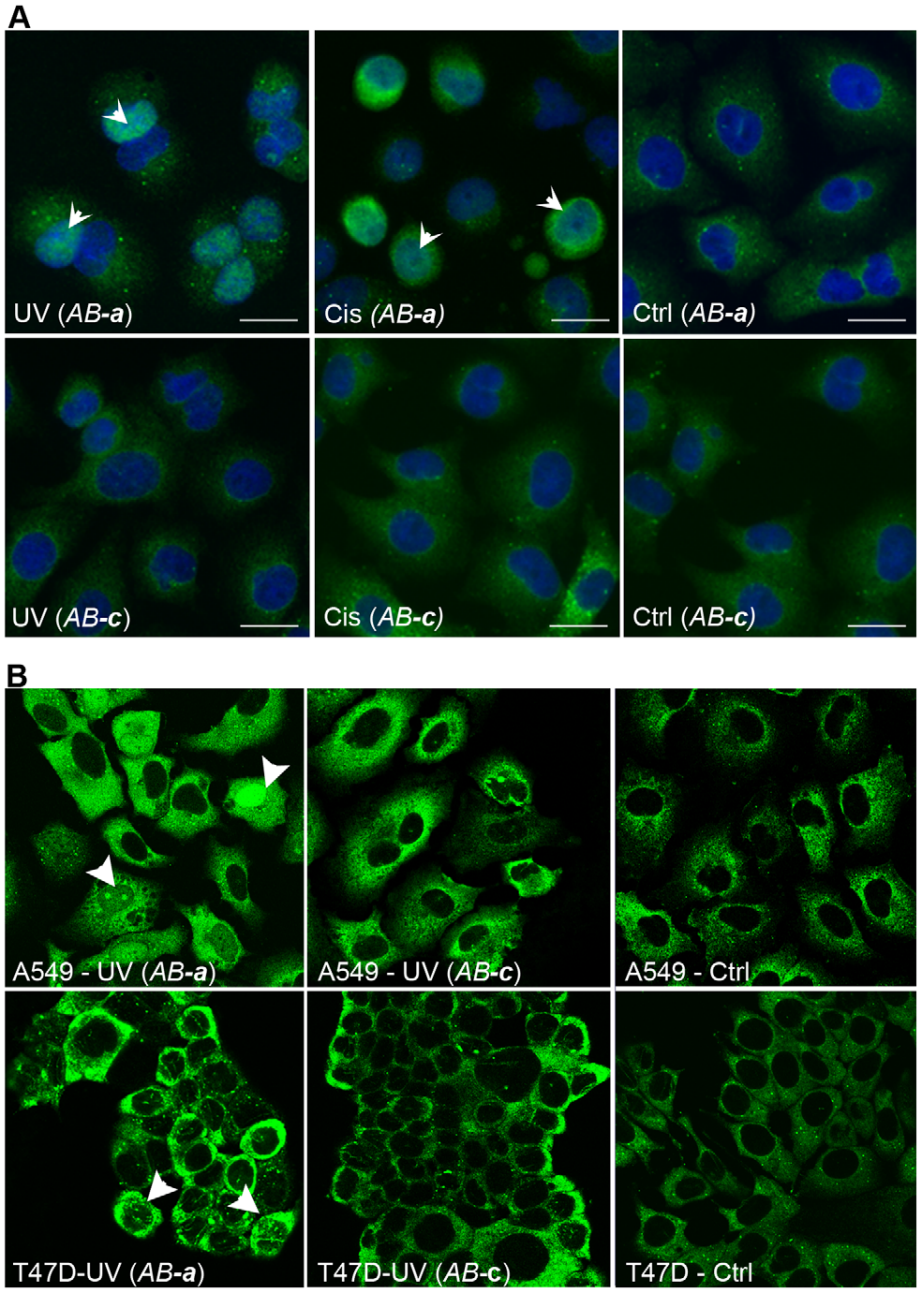
Stress induced nuclear translocation of YB-1 is detectable with antibody *AB-a.* **A**, Immunofluorescent analysis of YB-1 expression in A549 cells after ultraviolet (UV) light or cisplatin (Cis) treatment using antibodies *AB-* ***a*** and *AB-* ***c***. Cell nuclei are counterstained with DAPI that becomes translucent following YB-1 nuclear localization (merged image – see arrowheads). **B,** confocal immunofluorescent labeling of YB-1 levels using *AB-* ***a*** and *AB-* ***c*** in A549 and T47D cells after UV exposure. Arrowheads indicate nuclear YB-1. Ctrl = untreated controls.

As indicated above, nuclear YB-1 was rarely detected in tumours and only with *AB* ***-a***. A commonly reported feature of YB-1 is its propensity to translocate to the nucleus of cells after being subjected to treatment with different stressors [7], [9], [27]. To investigate whether the two antibodies can bind to nuclear YB-1, we increased the number of A549 cells with nuclear-localised YB-1 by treating with UV radiation and cisplatin. Results show that nuclear YB-1 was detectable with *AB-* ***a*** after treatment with UV and cisplatin (Figure 7A, arrowheads), but no nuclear YB-1 staining was observed in untreated control cells. In contrast, only cytoplasmic YB-1 staining was detected with *AB-* ***c*** irrespective of treatment. Similar results were obtained with MCF-7 breast cancer cells (Figure S3). We next used confocal immunofluorescence microscopy to discern whether this apparent nuclear YB-1 detected with *AB-* ***a*** was indeed nuclear or perinuclear as seen above. Using A549 cells, and a second breast cancer cell line, T47D, antibody *AB-* ***a*** showed clear punctuate and disperse nuclear localization (Figure 7B, arrowheads) and increased cytoplasmic intensity after exposure to UV radiation. However, no nuclear YB-1 was observed with *AB-* ***c***, although the cytoplasmic YB-1 component was readily detectable and the levels were also increased after stress treatment. Thus, as for the tumour IHC analysis, each YB-1 antibody displays a distinctive staining pattern that could lead to different interpretation. That is, based on *AB-* ***c*** staining, one could conclude that there is no stress induced nuclear translocation of YB-1, while the exact opposite conclusion could be derived from *AB-* ***a*** staining.

### Phosphorylation of epitope c does not prevent immunoreactivity of AB-c

**Figure 8:**
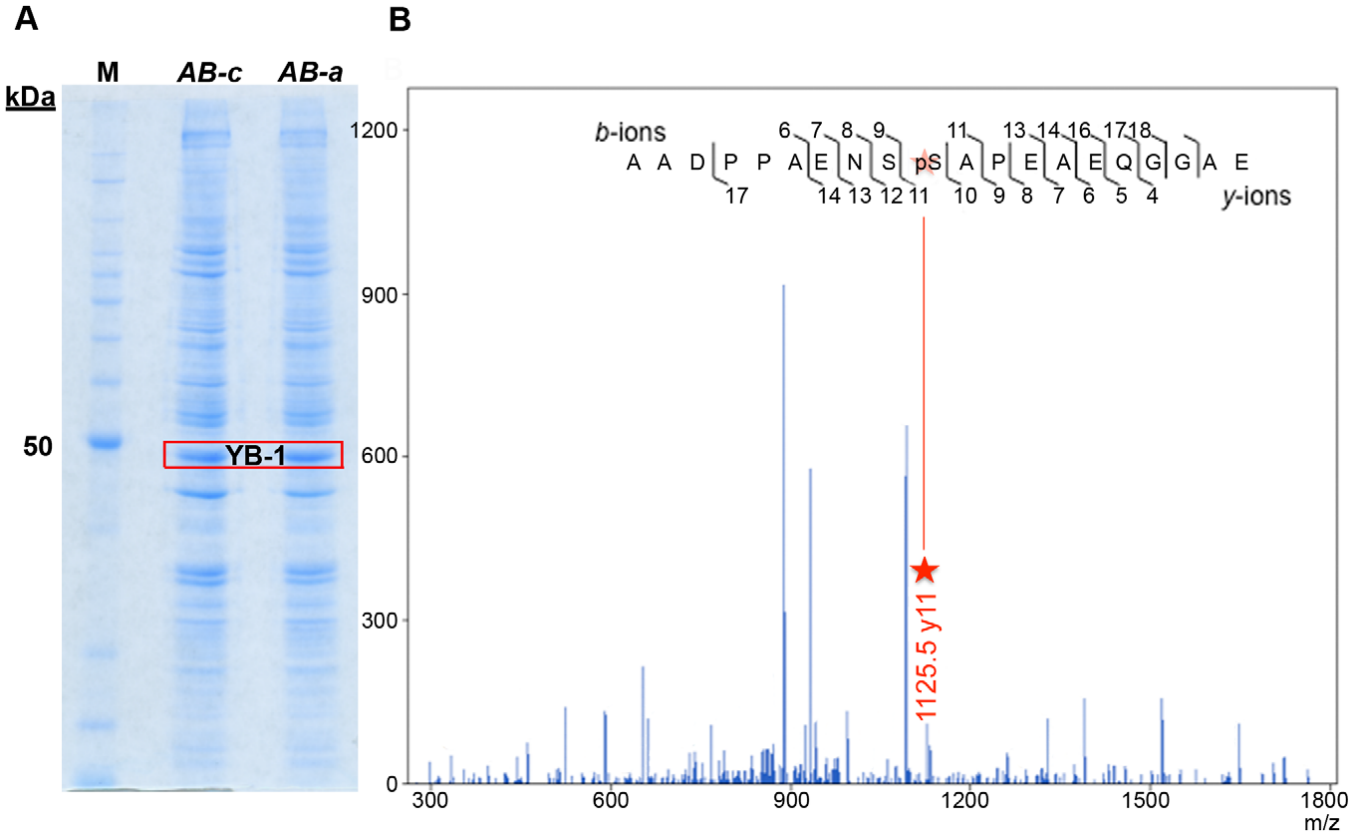
YB-1 Phosphoserine 314 is detected with both *AB-a* and *AB-c.* **A**, Coomassie-stained SDS–PAGE analysis of A549 cell extracts showing that YB-1 co-immunoprecipitates with either *AB-* ***a*** and *AB-* ***b.*** The bands contained within the red box were independently excised and subjected to LC/MS/MS analysis. **B**, A representative MS/MS spectra of the peptide AADPPAENSpSAPEAEQGGAE is shown. The observed *y*- and *b*-ions and the sequence of the peptide with the corresponding ions is shown. The data indicate phosphorylation of serine-314 (red star) and not serine-313.

**Figure 9:**
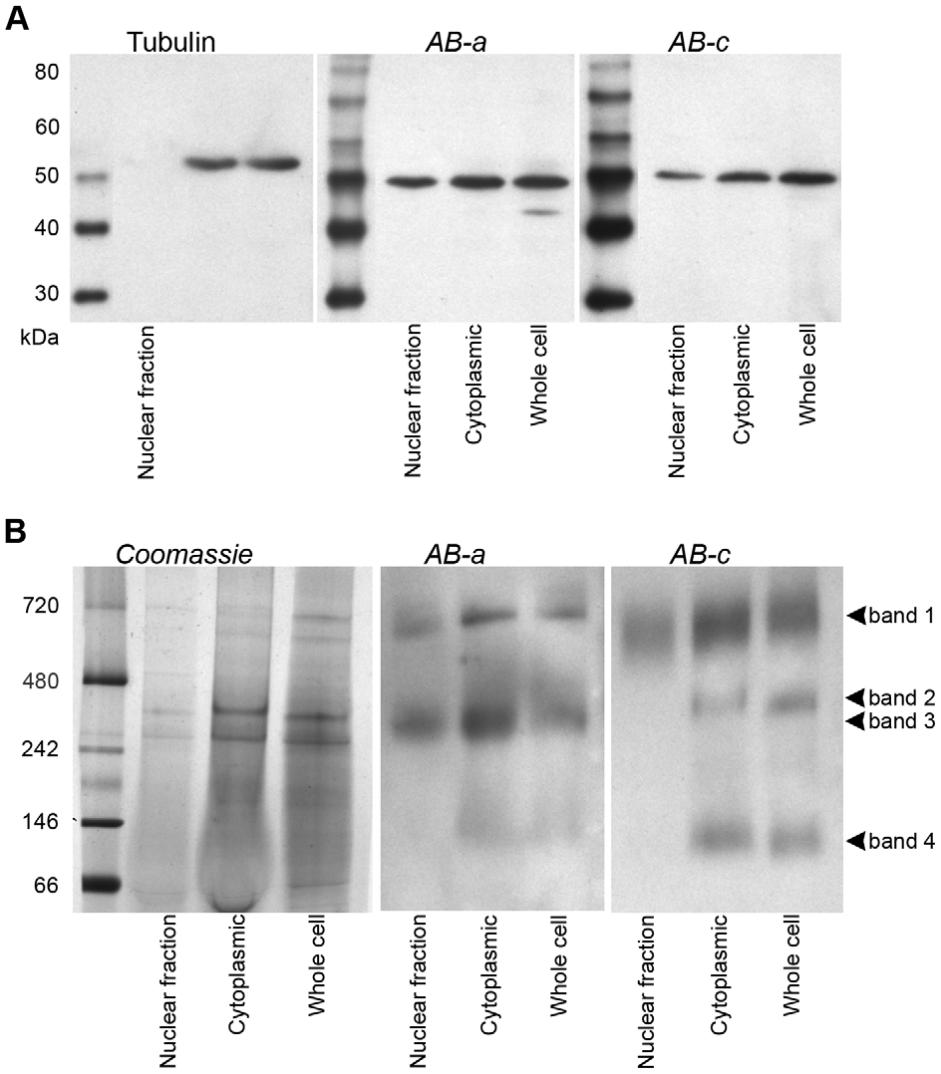
Different YB-1 antibodies detect different YB-1 protein complexes. **A**, nuclear and cytoplasmic fractions of A549 cells were subjected to SDS-PAGE and immunoblotting with antibodies *AB-* ***a*** and *AB-* ***c***. β-tubulin, exclusively cytoplasmic, was used as a control to confirm that the nuclear fraction did not contain any cytoplasm. **B**, A549 fractions from A were separated by BN-PAGE, immunoblotted and incubated with YB-1 antibodies *AB-* ***a*** and *AB-* ***c***.

One possible explanation as to why *AB-* ***c*** does not detect nuclear YB-1 and exhibits reduced sensitivity as a prognostic tool is that the epitope for *AB-* ***c*** is masked by post-translational modification(s). Phosphorylation of serine residues 313 and 314 have been reported [32], [33], [34], [35]. These are situated within, or directly adjacent to, epitope ***c*** (Figure 1) and where present may abrogate binding of *AB-* ***c*** but not *AB-* ***a***. To test this, A549 cells that had been treated with cisplatin were immunoprecipitated with either *AB-* ***a*** or *AB-* ***c*** (Figure 8A).**** Phosphopeptides generated from YB-1 tryptic digests were then subjected to LC/MS/MS. Phosphorylation of S314, but not S313, was detected in both samples derived from immunoprecipitation with *AB-* ***a*** and *AB-* ***c*** respectively, suggesting that phosphorylation of this residue does not interfere with the interaction of *AB-* ***c*** with YB-1 (Figure 8B). Thus, it seems unlikely that phosphorylation in the region of the epitope recognised by *AB*-***c*** could be a contributing factor in the reduced sensitivity observed in the breast tumours by *AB-* ***c***. Furthermore, western blotting of cell-line lysates with both *AB-* ***a*** and *AB-* ***c***, as exemplified by Figure 9A, revealed immunoreactivity in both cytoplasmic and nuclear fractions. This further reinforces that phosphorylation, or any denaturation-resistant post-translational modification, in the region of either epitope, does not contribute to the observed differential immunoreactivity of *AB-* ***a*** and *AB-* ***c*** in the tumour samples.

### AB-a and AB-c detect distinct YB-1 protein complexes

An alternative explanation as to why *AB-* ***c*** is not detecting nuclear YB-1 could be that epitope ***c*** is masked by proteins interacting with YB-1. In order to investigate this possibility, nuclear and cytoplasmic fractions from A549 cells were separated by native electrophoresis and immunoblotted with antibodies *AB-* ***a*** and *AB-* ***c***. Both antibodies detected YB-1 in multiple high molecular weight complexes (Figure 9B). While *AB-* ***a*** and *AB-* ***c*** detected YB-1 complexes of ≈720 kDa (band 1) in both nuclear and cytoplasmic fractions, the nuclear complexes detected by *AB-* ***a*** were more discrete in comparison to those detected by *AB* ***-c***. These data suggest that *AB-* ***a*** detects a sub-population of the YB-1 containing complexes that migrate at that mass. *AB-* ***c*** also detected two other YB-1 complexes of ≈300 kDa (band 2) and ≈100 kDa (band 4) in the cytoplasmic fraction that are both absent from the nuclear fraction. Band 4 corresponds to monomeric YB-1 and is weakly detected by *AB-* ***a***, as denatured YB-1 runs with the same mobility (data not shown). *AB-* ***a*** also detected a YB-1 complex of ≈250 kDa (band 3) in the nuclear and cytoplasmic fractions. That band 3 is detected in the nucleus by *AB-* ***a*** but not by *AB-* ***c*** would suggest that the nuclear staining observed in cells by immunofluorescence (and in the tumours) is associated with this complex. Presumably the band 1 complex (common to both antibodies) is masked under immunostaining conditions, at least in the nucleus. These findings are consistent with the interpretation that YB-1 forms discrete functional complexes and that epitope ***c*** of YB1 is not available for binding by *AB-* ***c*** in a subset of these functional complexes.

## Discussion

We set out to determine whether IHC analysis of breast tumours using antibodies generated to different YB-1 epitopes might lead to different conclusions about the value of YB-1 as a prognostic tool. Using two antibodies, we investigated whether there are differences in the subcellular localisation of YB-1 and whether its abundance increases with tumour progression. Two different tumour cohorts were used in this study, one from NZ and one from Singapore. With the N-terminal antibody (*AB-a*), we found that the staining intensity of cytoplasmic YB-1, although variable, tended to be highest in grade 3 tumours and in the more aggressive ER/PR negative tumours. These findings confirm previous reports, carried out with an antibody generated to the same N-terminal epitope [11], [19], [21]. The increase in staining was most marked in the cytoplasmic region directly adjacent to the nucleus (perinuclear), possibly due to an increase in YB-1 binding to RNA within the endoplasmic reticulum, as previously described [36]. However, unlike reports using an antibody generated against an N-terminal epitope [11], [19], [21], we detected few instances (<3%) of nuclear staining in any tumour. Further investigation by confocal immunofluorescence microscopy showed that *AB-**a*** detected punctate nuclear staining in grade 3 tumours which is not discernable by light microscopy and therefore previous reports may have interpreted the increased perinuclear intensity as nuclear staining due to the plane of section.

Cytoplasmic YB-1 detected with *AB-**c*** was also highest in grade 3 and ER/PR negative tumours. It did not reach statistical significance in the smaller NZ cohort but did show a significant difference in the Singapore cohort; however, this was of lesser magnitude than that found with *AB-**a***. As with *AB-**a***, the increase in cytoplasmic staining was most notable in the perinuclear region. These findings are in agreement with elevated YB-1 levels, detected by an antibody generated to the same C-terminal peptide sequence, previously reported in breast cancer [22], [24], non-small cell lung cancer [13] and prostate [12]. However, unlike some of these reports, our analysis with this antibody using confocal immunofluorescence microscopy did not find any evidence of nuclear YB-1 staining.

Notably, we found cytoplasmic YB-1 in all normal breast tissue, particularly in luminal cells with CAPSS. This is the first report that YB-1 is elevated in cells with CAPSS lesions, which have previously been reported to be ER/PR negative [37]. The increased level of YB-1 in these cells is consistent with our findings in tumour tissue where YB-1 is elevated in ER/PR negative tumours. The presence of YB-1 in normal breast tissue is in contrast to previous findings [11], [21].

The differences observed in immunoreactivity of *AB-**a*** and *AB-**c*** in tissues was examined further in tissue culture after treating cells with DNA damaging agents to induce nuclear translocation of YB-1. Again, we found that stress treatment increased YB-1 levels in the cytoplasm as detected with both antibodies but nuclear YB-1 was only detected with *AB-**a***. Thus, *AB-**a*** detected a sub-population of YB-1 molecules that *AB-**c*** did not.

To explain these differences a number of possibilities were considered. The most obvious was that the antibodies have different binding affinities to YB-1. However, the *K_d_* of each antibody, as measured by immunoprecipitation, was found to be approximately 5 nM (data not shown), making this explanation unlikely. It could be that *AB-**c*** is unable to access epitope ***c*** when YB-1 is located in the nucleus due to fixation procedures, as suggested previously [22], [24]. However, this also seems unlikely, as this antibody did not detect nuclear YB-1 with immunocytochemistry on cultured cells, where fixation and tissue thickness is not an issue. Another possibility is that epitope ***c*** is obscured by post-translational modifications that are important when YB-1 is in its native conformation. Phosphoserine 314, adjacent to epitope ***c***, was identified in both *AB-**c*** and *AB-**a*** immunoprecipitated YB-1. Therefore, phosphorylation of YB-1 on serine 314 seems to be an unlikely explanation. Nuclear YB-1 can be phosphorylated at serine 102 [38], [39] and it has been proposed that this phosphorylation is required for stress-induced nuclear translocation of YB-1. It is therefore possible that phosphorylation at serine 102 induces a conformational change to prevent the binding of *AB-**c***, however, serine 102 was not detected with either antibody in these experiments.

Another possible explanation for the apparent differences in epitope availability may be due to steric inhibition caused by protein-protein interactions. Protein lysates separated in the absence of reducing or denaturing agents to preserve protein:protein moieties, suggest that antibodies *AB-**a*** and *AB-**c*** differ in their ability to detect specific YB-1 containing protein complexes. The detection of YB-1 by *AB-**c*** in the nuclear fraction is contrary to our findings that *AB-**c*** is unable to detect nuclear YB-1 in either paraffin-embedded tissue or in cell culture. A reason for this discrepancy may be that YB-1 binds RNA to form homodimeric YB-1:RNA complexes [40]. The formation of these YB-1:RNA complexes involves the CSD and the C-terminal region [41] and may render epitope ***c*** inaccessible in YB-1 molecules that are integrated into the YB-1:RNA complexes. The role of the C-terminal region in forming these YB-1:RNA multimers increases as the YB-1:mRNA ratio increases [40]. Our IHC results correlated elevated YB-1 levels with grade and negative ER/PR status. Therefore, the increased sensitivity of *AB-**a*** relative to *AB-**c*** may be due to a reduction in the availability of epitope ***c*** caused by the effects of increasing YB-1:RNA ratio on YB-1:RNA multimer formation.

The data presented highlight the need for standardized antibodies for analysis of clinical material if YB-1 is to gain acceptance as a reliable prognostic marker, as has been done for the prognostic determination of the ER and HER2 immunostaining on breast cancers [42], [43]. Our results suggest that the staining pattern depends on epitope accessibility to the antibody and that apparent absolute levels are not in fact absolute; rather, they are levels of YB-1 available for detection, which in turn is dependent on the nature of the complex containing YB-1. From our data it would appear that antibodies to the extreme N-terminus of YB-1 seem to be the most sensitive because this region does not interact with other proteins to preclude recognition of its epitope, as appears to occur with *AB-**c***. Validated monoclonal antibodies to this region could ultimately be the best way of ensuring reproducibility, thereby realising the potential of YB-1 as a reliable marker of cancer progression and prognosis.

## Supporting Information

Figure S1 siRNA knockdown of YB-1 shows that cytoplasmic staining of YB-1 by *AB-* ***b*** is predominantly YB-1, whereas nuclear staining is hnRNP A1. **A**, Immunofluorescent staining with *AB* ***-b*** following knockdown with either siYB-1 or a non-targeting siRNA (NT) in both untreated (-), ultra violet treated (UV) and cisplatin-treated (Cis) cells****. Cytoplasmic staining is absent following knockdown with siYB-1. **B**, Western blot showing that YB-1, as detected by *AB-* ***b*** (50 kDa), is reduced following knockdown with siYB-1, hnRNP A1, as detected by *AB-* ***b*** (37 kDa), is reduced following knockdown with si hnRNP A1.
(TIF)

Figure S2 YB-1 is present in both immortalised and non-immortalised cell lines. The amount of YB-1 in a panel of immortalised and non-immortalised cell lines was determined by Western blotting using YB-1 antibody *AB-* ***a***. An antibody to β-tubulin (DSHB) was used as a loading control.
(TIF)

Figure S3 Stress induced nuclear translocation of YB-1 in MCF-7 cells is detectable with antibody *AB-* ***a*** but not *AB-* ***c***. Immunofluorescent analysis of YB-1 expression in MCF-7 cells after ultraviolet (UV) light or cisplatin (Cis) treatment using antibodies *AB-* ***a*** and *AB-* ***c***. Cell nuclei are counterstained with DAPI that becomes translucent (arrowheads) following YB-1 nuclear localization. Ctrl  =  untreated controls.
(TIF)
