# Supplementary figures and images for: Prognostic Association of YB-1 Expression in Breast Cancers: A Matter of Antibody
Source: PLoS One. 2011 Jun 10;6(6):e20603. doi: 10.1371/journal.pone.0020603 (PMC3112203; doi:10.1371/journal.pone.0020603)

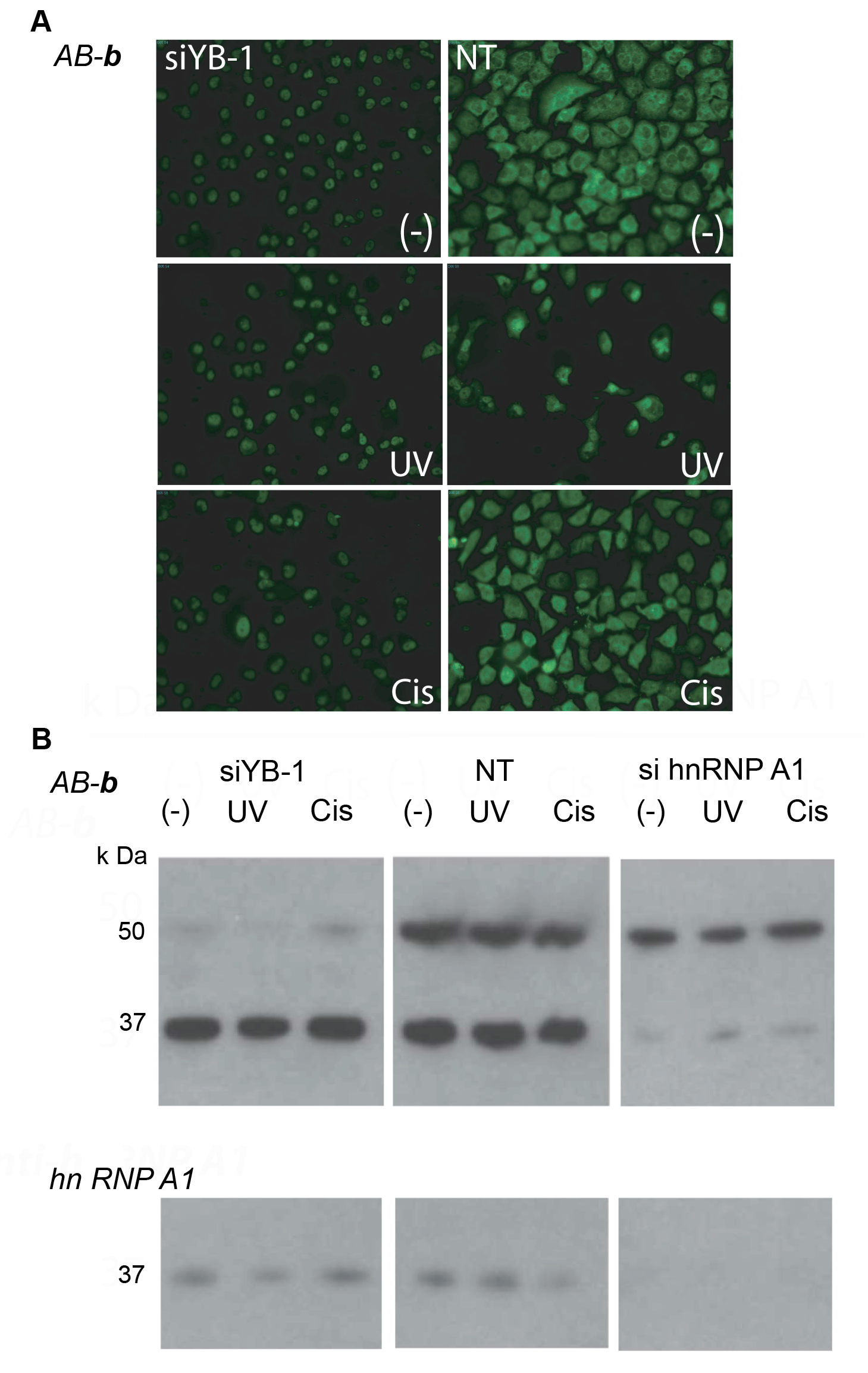

Supplement: Figure S1 — siRNA knockdown of YB-1 shows that cytoplasmic staining of YB-1 by AB- b is predominantly YB-1, whereas nuclear staining is hnRNP A1. A, Immunofluorescent staining with AB -b following knockdown with either siYB-1 or a non-targeting siRNA (NT) in both untreated (-), ultra violet treated (UV) and cisplatin-treated (Cis) cells. Cytoplasmic staining is absent following knockdown with siYB-1. B, Western blot showing that YB-1, as detected by AB- b (50 kDa), is reduced following knockdown with siYB-1, hnRNP A1, as detected by AB- b (37 kDa), is reduced following knockdown with si hnRNP A1. (TIF) [file pone.0020603.s001.tif]

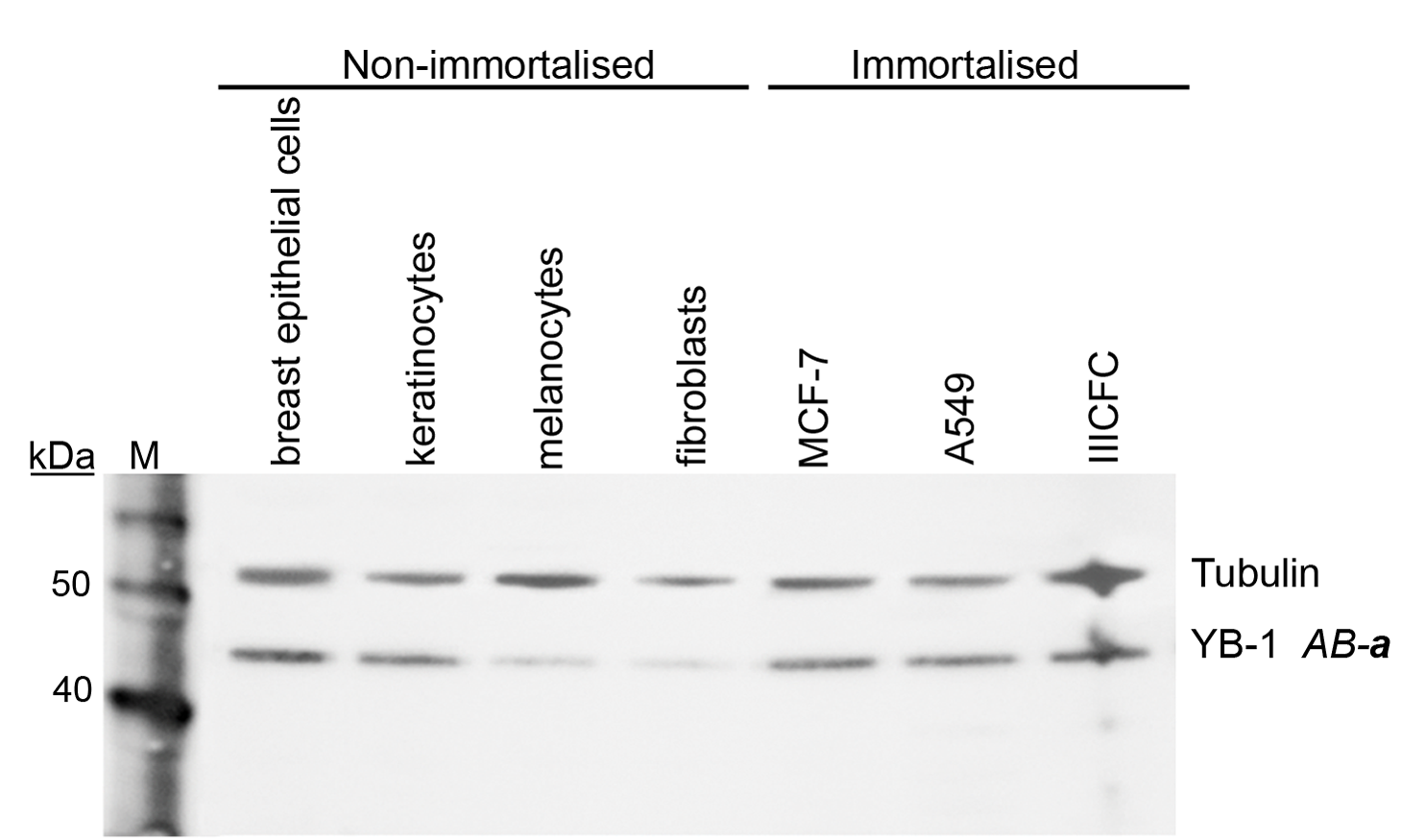

Supplement: Figure S2 — YB-1 is present in both immortalised and non-immortalised cell lines. The amount of YB-1 in a panel of immortalised and non-immortalised cell lines was determined by Western blotting using YB-1 antibody AB- a. An antibody to β-tubulin (DSHB) was used as a loading control. (TIF) [file pone.0020603.s002.tif]

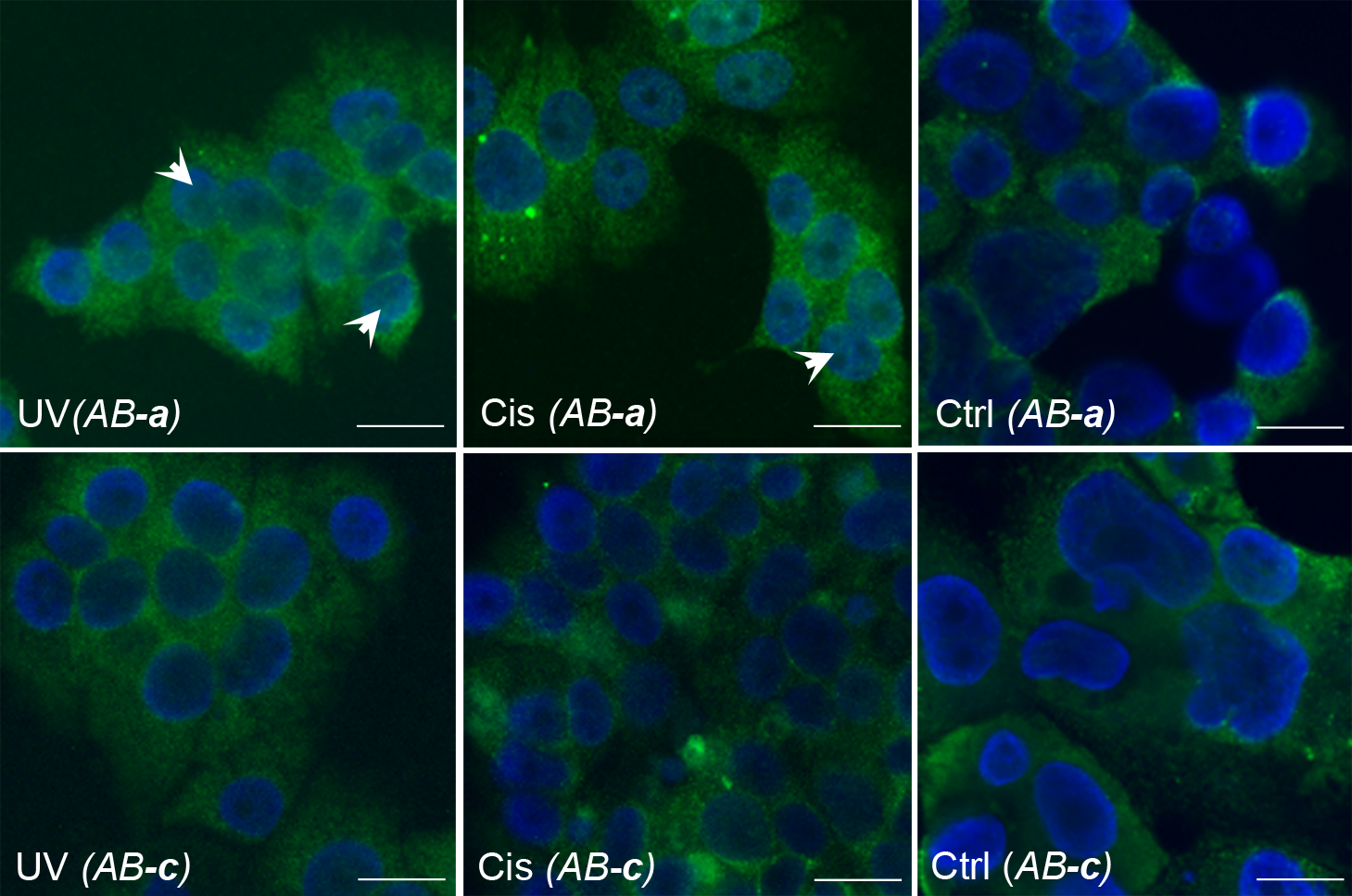

Supplement: Figure S3 — Stress induced nuclear translocation of YB-1 in MCF-7 cells is detectable with antibody AB- a but not AB- c. Immunofluorescent analysis of YB-1 expression in MCF-7 cells after ultraviolet (UV) light or cisplatin (Cis) treatment using antibodies AB- a and AB- c. Cell nuclei are counterstained with DAPI that becomes translucent (arrowheads) following YB-1 nuclear localization. Ctrl = untreated controls. (TIF) [file pone.0020603.s003.tif]
